# Effect of Common Cooking and Drying Methods on Phytochemical and Antioxidant Properties of *Corchorus olitorius* Identified Using Liquid Chromatography-Mass Spectrometry (LC-MS)

**DOI:** 10.3390/molecules27249052

**Published:** 2022-12-19

**Authors:** Buyile Ncube, Bhekisisa Dlamini, Daniso Beswa

**Affiliations:** Department Biotechnology and Food Technology, Faculty of Science, University of Johannesburg, Doornfontein Campus, P.O. Box 17011, Johannesburg 2028, South Africa

**Keywords:** *Corchorus olitorius*, TPC, TFC, antioxidant activity, cooking, drying, frying

## Abstract

In this study, *Corchorus olitorius* leaves were subjected to different thermal treatments (blanching, boiling, drying, frying, and steaming) and analyzed, total phenolic content (TPC), total flavonoid content (TFC), and antioxidant activity. Furthermore, Fourier transform infrared spectroscopy (FTIR) was used to identify functional groups, while metabolites were identified with LC-MC. The TPC and antioxidant activity of *C. olitorius* were significantly (*p* < 0.05) increased by cooking and drying. The steam-cooked sample had the highest TPC (18.89 mg GAE/g) and TFC (78.42 mg QE/g). With ABTS, FRAP, and DPPH assays, the steam-cooked sample exhibited the highest antioxidant activity of 119.58, 167.31, and 122.23 µM TE/g, respectively. LC-MS identified forty-two (42) metabolites in *C. olitorius* that included phenolic acid derivatives, flavonoid derivatives, and amino acid derivatives. Overall, steaming appears to be the best cooking method, with respect to the retention of phytochemical compounds and antioxidant activity.

## 1. Introduction

Indigenous African leafy vegetables (ALVs) play a significant role in the daily food preparation of many low-income households in sub-Saharan Africa (SSA). Generally, SSA is plagued by a food security crisis that encourages dependency on economical food options, such as ALVs, to bridge the gaps in nutrition, especially during off-season and famine. In addition to being a relief food commodity, ALVs are high in compounds that possess antioxidant activity known to be beneficial to consumer health. This has prompted an increasing interest in the consumption of plant-based diets among health-conscious consumers, such as teenagers and youth [1,2]. This interest is also incited by the increased number of individuals leading a sedentary lifestyle and the fact that they are readily available and cheap [3].

The phenolic compounds and bioactive nutrients present in ALVs are known to provide the consumer with desirable health benefits beyond basic nutrition [4,5]. These health benefits are observed after regular consumption of vegetables. According to Cencic and Chingwaru [6] and de Lima et al. [7], there is a link between regular consumption of vegetables and lower risks of chronic ailments, such as cancer, hypertension, diabetes, obesity, dyslipidemia, and cardiovascular and respiratory diseases. Some ALVs are rich in heat-sensitive vitamin C, water-soluble Vitamin A, zinc, and iron. *C. olitorius* is more popular in tropical climates worldwide (Asia, America, and Africa). It grows extensively in the eastern and northern regions of South Africa, including Kwazulu-Natal, Limpopo, the Eastern Cape, and Mpumalanga [8]. This leafy vegetable is rich in vitamins (C, E, and A), minerals (iron, calcium, and magnesium) [9,10], and its leaves are rich in antioxidant compounds linked with a variety of biological characteristics [11]. As a consequence, its leaves, stems, and roots are frequently used in human nutrition and traditional medicine formulation [12]. The leaves have a high concentration of antioxidant compounds linked to a wide range of biological activities [9,10,11]. In order to reap the benefits of this plant, its leaves, stems, and roots are incorporated into many human diets and utilized in the manufacture of traditional medicine [12]. The cooked leaves are used to increase women’s and children’s immunity, alleviate blood loss in new mothers, and heal injuries [13]. Seeds are used as a laxative [14] while root infusions are used to cure chest pains, gonorrhea, and toothaches [15,16]. As *Corchorus olitorius* has remarkable health potential benefits linked to phenolic compounds and their derivatives, it is vital to investigate how cooking and drying would alter the vegetable. For this research, the total phenolic content, total flavonoid content, and free radical scavenging activity were determined after cooking and drying. The full metabolite profile of *C. olitorius* is explored as well after different cooking and drying methods to determine to what extent cooking and drying affects each individual metabolite.

## 2. Materials and Methods

### 2.1. Chemicals

All reagents used were of analytical or HPLC grade. Aluminum chloride, quercetin, nitric acid, acetic acid, hydrochloric acid, Sodium carbonate, acetic acid, hydrochloric acid, Sodium nitrite, 6-Hydroxy-2,5,7,8-tetramethylchoman-2-carboxylic acid (Trolox), 2,20-azinobis (3-ethylbenzothiazoline-6-sulfonic acid) diammonium salt (ABTS) radical, Sodium acetate trihydrate, Folin–Ciocalteu reagent, Sodium nitrite, Sodium hydroxide, methanol, 2,4,6-tripyridyl-s-triazine (TPTZ), acetic acid, and gallic acid were utilized.

### 2.2. Sample Preparation

Fresh *C. olitorius* leaves were purchased from *Tshimbupfe* village, Limpopo province (South Africa). The leaves were packed in sterile polyethylene bags and transported under cool conditions (in a cooler box containing ice) to the laboratory, washed with cold water, and processed within 24 h of harvesting. A method by Lewu et al. [17] involved 100 g of leaf sample being added to 200 mL of boiling water and cooked for 10 min. The boiled vegetables were removed from the water and allowed to cool in the pot to room temperature. A method by Moyo et al. [4] and Musa and Ogbadoyi [18] with slight modifications was employed for drying: 100 g vegetable sample was oven-dried at 65 °C for 5 h. Blanching was performed according to a method by Moyo et al. [4] with slight modifications; a 100 g leaf sample was blanched at 82 °C for 5 min and rapidly cooled using cold water. According to Adefegha and Oboh [19] and Moyo et al. [4], a steaming method involved a 100 g sample being steamed using a steam basket at 90 °C for 5 min. According to Traoré et al. [1], a frying method involved a 100 g sample being fried at 120 °C for 5 min using olive oil and another 100 g being fried using vegetable oil. All processed samples were freeze-dried until further analysis.

### 2.3. Solvent Extraction

Two grams of leaf was ground, after which it was mixed with 20 mL of an 80% methanol solution. Sonication of the mixture for 10 min using an ultrasonic sonicator was conducted, after which centrifugation for 10 min at 3000 rpm followed with a temperature of 4 °C being maintained. Using a Buchi rotary evaporator, the mixture was evaporated until 1 mL was yielded. The extracts were covered with foil paper and allowed to dry in a dry, cool environment for 24 h, after which they were kept at −20 °C until analysis.

#### 2.3.1. Determination of Total Phenolic Content

The Folin–Ciocalteau (F-C) method, defined by Ainsworth and Gillespie [20], was used to approximate total phenolic content (TPC) of the extract. F-C reagent was diluted 15 times using deionized water. The gallic acid standard curve (0–0.2 mg/mL) was prepared in 21 1.5 mL Eppendorf tubes for calibration curve in triplicates. An amount of 10 µL of sample was combined with 50 µL F-C reagent and 50 µL sodium carbonate (Na_2_CO_3)_ into the 96-well microplate (flat bottom wells type). Absorbance at 750 nm was determined using an iMark microplate reader (Bio-Rad laboratories, Inc, Hercules, CA, USA).

#### 2.3.2. Determination of Total Flavonoid Content

The Aluminum chloride method by Al- Farsi and Lee [21] was used to approximate the total flavonoid content (TFC) of extract. A quercetin standard curve (0–2 mg/mL) was prepared in 21 1.5 mL Eppendorf tubes for calibration curve triplicates. An amount of 10 µL of sample, followed by 30 µL AlCl_3_, and lastly 100 µL NaOH were added into the 96-well microplate (flat bottom type). An iMark microplate reader (Bio-Rad laboratories 168–1130) was utilized to read absorbance at 450 nm and results were expressed as mg Quercetin equivalents/g.

### 2.4. Determination of Antioxidant Activity

#### 2.4.1. ABTS Radical Scavenging Activity Assay

The ABTS -2, 2-azinobis (3-ethyl-benzothiazoline-6-sulfonic acid) test described by Awika et al. [22] was utilized to determine antioxidant activity. Equal amounts of 8 nM ABTS+ and 3 nM potassium sulphate were combined, and the solution was incubated for 12 h at room temperature and utilized within 16 h. A 0.26 nM solution of the ABTS radical was produced by further diluting ABTS stock 30 times using 0.1 M PBS. An amount of 10 µL vegetable extracts were pipetted into a 96 well microplate followed by 290 µL of the ABTS free radical cation solution. Incubation in the dark at 37 °C for 15 min was conducted. Readings were measured at 750 nm via iMark microplate reader (Bio-Rad laboratories 168–1130).

#### 2.4.2. DPPH Radical Scavenging Activity Assay

A method by Awwad et al. [23] was used to approximate the DPPH-(2, 2-diphenyl-1-picrylhydrazyl) assay. Six hundred micromolar (600 µM) DPPH (0.024 g DPPH in 100 mL methanol) was prepared and then 10 mL of stock solution was diluted using methanol. Incubation in the dark was done for 20 min. An amount of 15 µL of sample and 285 µL of DPPH solution were placed in a 96 well microplate (flat bottom) and incubated at 37 °C for 15 min. Readings were measured at 570 nm using an iMark microplate absorbance reader (Bio-Rad laboratories 168–1130).

#### 2.4.3. Ferric Reducing Antioxidant Power Assay (FRAP)

The ferric reducing ability of plasma (FRAP) assay test, described by Adedapo et al. [24], was used. A working solution was prepared by combining 2.5 mL of 2,4,6-tripyridyl-s-triazine (TPTZ), 3 mL distilled water, 2.5 mL iron chloride, and 25 mL acetate buffer. Water bath temperature was allowed to reach 37 °C before use. An amount of 30 µL of the sample were pipetted together with 900 μL of FRAP solution into a test tube. This was stored in the dark for 30 min, after which readings were taken at 595 nm using an iMark microplate reader.

### 2.5. Fourier Transform Infrared Spectroscopy (FTIR)

All cooked and dried samples were analyzed using an FTIR spectrophotometer (Thermo Scientific Smart iTR, (Attenuated Total Reflectance), Thermo Fisher Scientific Inc., Waltham, MA, USA). Prior to placing samples, 0.2 g of crushed leaf powder, the instrument was cleaned with 70% ethanol and background spectra were acquired. Each sample’s spectrum was captured at 16 runs per scan in the range of 500 cm^−1^ to 4000 cm^−1^, which corresponds to the distinctive peak range of phenolic functional groups. The Origin data analysis software was used to plot FTIR data.

### 2.6. Extraction of Metabolites

Plant extracts were obtained by use of 80% methanol/water (*v*/*v*) (4:1 ratio). A method described by Abu-Reidah et al. [25] and Ramabulana et al. [26] with slight changes was employed for extraction. Two grams of ground *Corchorus olitorius* leaves were mixed with 20 mL of 80% methanol and sonicated using an ultrasonic sonicator at room temperature for 15 min. The resultant mixture was centrifuged at 5000 rpm for 10 min and subsequently evaporated using a Buchi rotary evaporator to approximately 1 mL. The extracts were further dried to completeness overnight at 4 °C. Methanol (80%) was used again to dissolve the extracts, and filtration through a 0.22 µm syringe filter was conducted. The filtrate was stored at −20 °C until analysis.

### 2.7. Analysis Using Liquid Chromatography-Mass Spectrometry (LC-MS)

The leafy vegetable samples that were processed by boiling, drying, blanching, frying with olive oil, and steaming were selected for further analysis using LC-MS because they showed better outcomes with respect to proximate composition, TPC, TFC, and antioxidant activity (ABTS, FRAP, and DPPH assays). For LC-MS analysis, a Waters Synapt G2 Quadrupole time-of-flight (QTOF) mass spectrometer (MS) was linked to a Waters Acquity ultra-performance liquid chromatography (UPLC) (Waters, Milford, MA, USA). UV and MS spectra was done by passing the column eluate through a Photodiode Array (PDA) detector. Negative electrospray ionization was employed with a cone voltage of 15 V, desolvation temperature of 275 °C, desolvation gas at 650 L/h, and the remainder of the MS parameters tuned for optimal resolution and sensitivity. Data were collected by scanning in resolution mode and MSE mode from 150 *m*/*z* to 1500 *m*/*z*.

The MSE mode had two channels, namely collision energy ramp of 40–100 V and another of 4 V. This was used to obtain fragmentation data. Leucine enkaphalin was employed as reference mass; calibration was performed using sodium formate. A Waters HSS T3, 2.1 100 mm, 1.7 m column was used to separate the samples. Solvent A, which was the mobile phase, was made up of 0.1% formic acid and acetonitrile containing 0.1 percent formic acid was solvent B, with a total injection volume of 2 L. The gradient began with 100 percent solvent A for 1 min and then shifted to 28 percent B in a linear fashion over 22 min. It then moved to 40 percent B for 50 s, then 1.5 min at 100 percent B, followed by 4 min of re-equilibration to original conditions. The column temperature was maintained at 55 °C and the flow rate was 0.3 mL/min. The different catechin standards (0.5–100 mg/L) formed the calibration curve, which, in turn, was used to determine compounds [27,28].

### 2.8. Statistical Analysis

All experiments were conducted in triplicate and the data were analyzed using Statistical Package for the Social Sciences (SPSS) version 21 (2018) for Windows (SPSS IBM, New York, NY, USA). One way analysis of variance (ANOVA) and mean comparison was done using Duncan’s multiple range tests. Mean values were considered significantly different at *p* < 0.05. Results obtained were expressed as the mean values ± the standard deviation. The LC-MS data for the concentration of identified metabolites were subjected to principal component analysis (PCA) using OriginPro 2022b (OriginLab, Northampton, MA, USA).

## 3. Results and Discussion

### 3.1. Effect of Common Cooking and Drying Methods on Total Phenolic and Flavonoid Content

The effect of common cooking and drying methods on the TPC and TFC of *Corchorus olitorius* leaves are shown in Table 1. Generally, cooking and drying *C. olitorius* leaves improved the TPC and TFC, with boiling being an exception in regard to TPC. The steam-cooked leaves exhibited a higher TPC (118.89 mg GAE/g) than the control and other cooking methods. Adefegha and Oboh [19] reported that an increase in TPC and TFC could be due to minimal nutrient leaching that occurs during steaming, since the vegetable has no contact with boiling water. There was no significant difference (*p* < 0.05) in TPC between the control and boiled sample. The control sample depicted a TPC of 47.40 mg GAE/g. The obtained result is higher than that observed by Youssef et al. [16] of 16.54 ± 0.63 mg GAE/g but lower than that obtained by Andabati and Muyonga [29] (62.3 mg GAE/g). This discrepancy might be associated with differences in climatic conditions, which could impact the phenolic compound composition of the plant, as well as the method of extraction utilized. The steamed sample yielded the highest TFC (78.42 mg QE/g). This might be due to the decreased nutrient leaching that occurs during steaming, since there is no contact between the vegetable and boiling water [19]. Furthermore, the heat from the steam might promote the elimination of phenolic compounds from proteins found inside cells and cell wall components [4]. Adefegha and Oboh [19] and Thi and Hwang [30] observed that steaming enhances the TFC. In this research study, it was observed that boiling increased the TFC of *C. olitorius* leaves to 39.47 mg QE/g. The release of protein-bound flavonoids and dietary fibers may be facilitated by boiling, leading to an increase in the quantity of unbound flavonoids present in the vegetable material [31].

### 3.2. Antioxidant Activity

The antioxidant activity of *C. olitorius* leaves was determined by the DPPH, FRAP, and ABTS assays (Table 2). The steam-cooked samples displayed the highest antioxidant activity with DPPH (119.58), FRAP (167.31), and ABTS (122.23), in terms of µM TE/g. With DPPH and FRAP, the boiled sample depicted the lowest antioxidant activity of 56.70 and 72.62 µM TE/g, respectively. Among the assays, FRAP recorded the highest antioxidant activity, probably because *C. olitorius* has high reducing power. According to Craft et al. [32], reducing power is dependent on the conjugation of phenols and the quantity of existing hydroxyl groups. Cooking and drying increased the antioxidant activity of raw *C. olitorius* leaves in all assays conducted with moderate reductions in the DPPH and FRAP assays, with respect to the boiled sample.

After boiling, antioxidant activity decreased for spinach (*Spinacia oleracea*), as was reported by Mazzeo et al. [33] and Kunyanga et al. [34] for drumstick tree (*Moringa oleifera*) and pumpkin leaves. It is possible that the decreases are due to the antioxidant compounds leaching into the boiling water. Across all antioxidant assays, the steamed sample exhibited the highest antioxidant activity. This occurrence is in line with the findings of Adefegha and Oboh [19], Mazzeo et al. [33], and Miglio et al. [35] who reported an increase in antioxidant activity after green leafy vegetables were steamed.

Steaming induces the extraction of antioxidants from plant cell components while preventing the chemicals from leaching. Across all antioxidant assays, the vegetable material cooked in olive oil demonstrated relatively high antioxidant activity, in comparison to the other cooking and drying methods used. According to Tuck and Hayball [36], olive oil contains at least 30 phenolic compounds, hence considerable antioxidant activity.

### 3.3. Fourier Transform Infrared Spectroscopy (FTIR)

**Figure 1:**
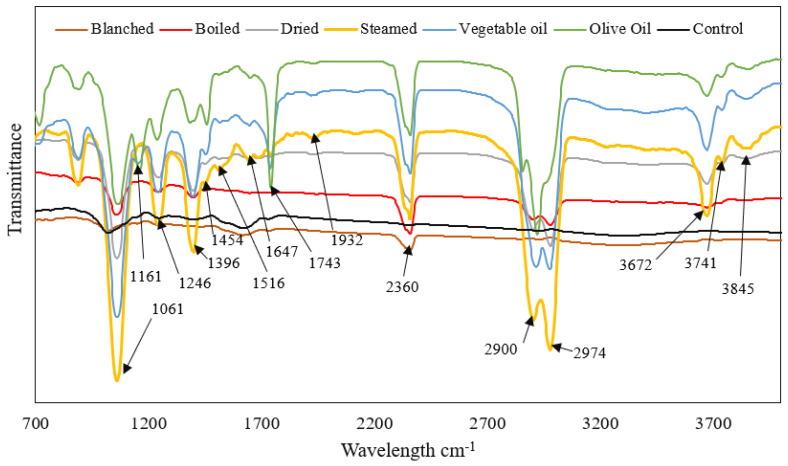
FTIR spectrum for *C. olitorius* after different cooking and drying methods. Blanched−blanched 82 °C/5 min, Dried−dried 65 °C/5 h, Boiled−boiled 100 °C/10 min, Steamed−steamed 90 °C/5 min, Vegetable oil−fried vegetable oil 120 °C/5 min, Olive oil−fried olive oil 120 °C/5 min.

The FTIR spectrum for *C. olitorius* after different cooking and drying methods were employed is shown in Figure 1. Bands in the 3845 cm^−1^, 3741 cm^−1^, and 3672 cm^−1^ could be due to the H-bonded hydroxyl group (OH). This functional group is usually visible in the 4000–3120 cm^−1^ region [37,38]. The O-H band designates the presence of phenolic groups and alcohols [23]. This aforementioned band is sharper for the steamed *C. olitorius* leaves. This is congruent to the results shown in Table 1 of the high total phenolic content exhibited by the sample. Peaks at 2974 cm^−1^ and 2900 cm^−1^ could be assigned to the alkenes functional group. The absorption band at 1932 cm^−1^ could be assigned to the aromatic ring or C=C, which has several weak overtones [37].

Absorption at 1743 cm^−1^ could be assigned to an ester bond, which manifests as a very strong and sharp band due to the C=O. The absorption band at 1647 cm^−1^ could possibly represent the presence of conjugated carbonyl bonds from flavonoids or hydroxyl groups [23,37]. The bands of 1516 cm^−1^ could be due to the C=C, which is weak. Absorption in the 1410 cm^−1^ and 1310 cm^−1^ region are typical of phenols’ OH functional groups. According to Coates [37], peaks in the 1170 cm^−1^–930 cm^−1^ may be due to the flavonoids and polysaccharide groups. The sharp absorption at 1061 cm^−1^ exhibited the lowest transmission and the band is sharpest for the steamed leaves. This band corresponds with the results shown in Table 1, implying that the steamed leaves had the highest total flavonoid content. Bands occurring at 930–700 cm^−1^ may be assigned to aromatic rings due to C-H bending [38,39].

### 3.4. Quantification and Qualification of Metabolites Using LC-MS

**Figure 2:**
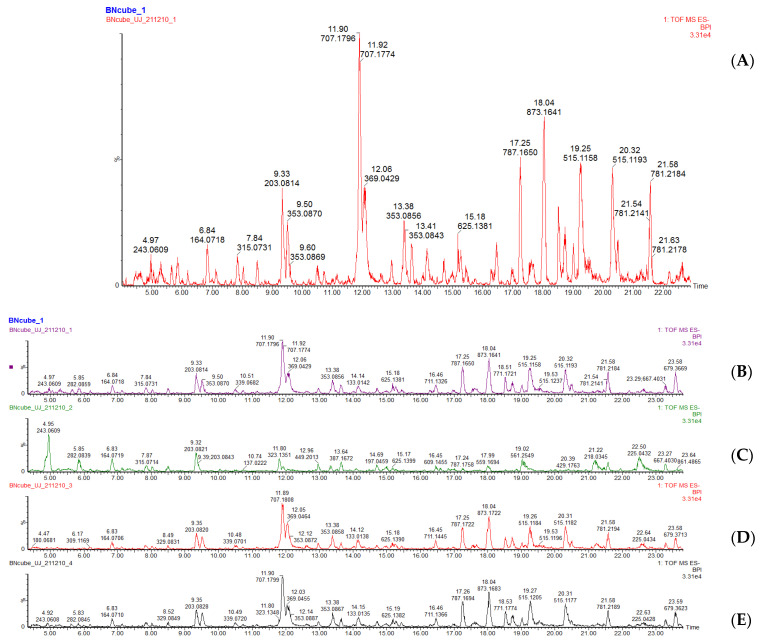
LC-MS chromatograms for samples (**A**) control, (**B**) blanched, (**C**) dried, (**D**) fried with olive oil, (**E**) steamed.

Plant metabolites play an essential role in the prevention of oxidative damage, and they are widely used for their biological activity. Boiling, drying, blanching, and frying with olive oil were selected for further analysis because they showed better outcomes in respect to proximate composition, TPC, TFC, and antioxidant activity. The LC-MS results from samples extracted with 80% methanol are shown in Figure 2. In addition, Table 3 presents a list of compounds detected from the LC-MS analysis. The LC-MS revealed the presence of 42 metabolites belonging to different metabolic groups, including phenolic acid derivatives, flavonoid derivatives, and amino acid derivatives.

#### 3.4.1. Characterization of Amino Acids

Amino acids are organic compounds made up of a basic amino group (-NH_2_), an acidic carboxyl group (-COOH), and an organic R group (side chain). The amino acid groups identified in all samples include L-phenylalanine (peak 1, *m*/*z* 164) and L-tryptophan (peak 6, *m*/*z* 203) (Figure 2). L-tryptophan in the control sample was the highest (285.3 mg/kg) compared to the other processing methods (0.4–278.1 mg/kg). Ito et al. [40] reported a decrease in L-tryptophan in spinach leaves after cooking in temperatures above 100 °C as the amino acid is destroyed by heat but the time–temperature combination for the dried sample ‘preserved and enhanced’ metabolic reactions that caused an increase in the amino acid concentration; hence, the dried sample had the second highest L-tryptophan concentration. Both these compounds (L-tryptophan and L-phenylalanine) are essential amino acids, meaning the human body cannot synthesize them. L-tryptophan is critical in numerous metabolic functions, such as protein, serotonin, tryptamine, melatonin, and niacin synthesis [41]. L-phenylalanine is vital in the production of epinephrine, dopamine, and nor-epinephrine, which are neurotransmitters. It is also essential for the proper functioning of the nervous system [42].

#### 3.4.2. Characterization of Phenolic Acids

Two types of phenolic acids were detected, which are hydroxybenzoic acid and hydroxycinnamic acid derivatives. The hydroxybenzoic acids detected included 4-hydroxybenzoic acid, also known as p hydroxybenzoic acid (PHBA) (peak 10, *m*/*z* 137) and syringic acid (peak 23, *m*/*z* 197). Cooking and drying thermally degraded PHBA. The control had the highest PHBA concentration (71.6 mg/kg) while the blanched sample had the highest syringic acid concentration 79.1 mg/kg. Syringic acid is used as a therapeutic agent in various diseases (diabetes, cancer, neuro, and liver damage). It also exhibits antimicrobial, anti-inflammatory, and antiendotoxic activities [43].

The hydroxycinnamic acids identified were chlorogenic acid (peak 18, *m*/*z* 353), crypto-chlorogenic acid (peak 14, *m*/*z* 353), caffeoylmalic acid (peak 22, *m*/*z* 295), neo-chlorogenic acid (peak 7, *m*/*z* 353), 4 hydroxycinnamic acid (peak 12, *m*/*z* 163), and caffeic acid (peak 16, *m*/*z* 179). Cooking and blanching improved the crypto-chlorogenic acid content of the leaves. The blanched, fried, and steamed samples depicted crypto-chlorogenic acid as the most dominant phenolic acid with concentrations of 601, 563.6, and 580.8 mg/kg, respectively. The control had the lowest phenolic acid concentration of 65.2 mg/kg. Crypto-chlorogenic acid (CGA) is one of the most available acids amongst phenolic acid compounds. It is vital and biologically active dietary polyphenol, playing several important and therapeutic roles, such as antioxidant activity, anti-inflammatory, antibacterial, antipyretic, anti-obesity, neuro-protective, anti-viral, free- radical scavenger, antimicrobial, and central nervous system stimulator [44].

The esterification reaction of cinnamic acid (CA) and quinic acid (QA) derivatives, including ferulic, sinapic, and caffeic acid, result in chlorogenic acids, which are a type of phenolic acids [45]. Three peaks were identified at 353 *m*/*z* region isomers of chlorogenic acid in all samples. The presence of chlorogenic acid and its isomers in *C. olitorius* leaves has been reported by Azuma et al. [46], Yakoub et al. [47], and Guzzetti et al. [48].

In this study, cooking improved the concentration of chlorogenic acid in the leaves. The blanched sample had the highest content (196.2 mg/kg) whilst the dried had the lowest concentration (6.7 mg/kg). According to Ramirez-Anaya et al. [49] and Nicoli et al. [50], an increase in chlorogenic acid was observed after cooking *Solanum melongena.* This could be due to the isomerization and hydrolysis reactions and redistribution of phenolic acid concentration due to massive trans-esterification phenomenon occurring during processing. On the contrary, Guzzetti et al. [48] reported a decrease in chlorogenic acid and its derivatives after cooking *C. olitorius* leaves, which is what was portrayed in this study after drying the leaves. Such a decrease could be due to heat degradation of the cell wall that then exposes the phenolic compounds, thus increasing their susceptibility to oxidative degradation [51].

Caffeic acid possesses anti-inflammatory and antioxidant properties. Furthermore, it can be employed in the prevention of cancer, diabetes, and neurodegenerative diseases [52]. Throughout all the treatment methods, there were low concentrations of caffeic acid (4.1 mg/kg to 71.7 mg/kg) with the fried sample exhibiting the highest concentration.

Dicaffeoylquinic acids (DCQA) were identified with a precursor ion 515 *m*/*z*, molecules 38–41. According to the literature, dicaffeoylquinic acids have a mass-to-charge ratio of 515.1463 *m*/*z* (C_25_H_23_O_12_) [53]. The 3.5 dicaffeoylquinic acid in *C. olitorius* leaves has been reported by Azuma et al. [46]. The blanched sample exhibited the highest concentration of 398.1 mg/kg compared to other processing methods (44.5–379.8 mg/kg), with the dried sample exhibiting the lowest concentration. The heat denaturation of plant enzymes, such as peroxidases, glucosidases, and polyphenol oxidases, which are responsible for the oxidation of phenolic compounds, might account for an increase in dicaffeoylquinic acid content after blanching. The denaturation of these enzymes might have prevented the oxidation of dicaffeoylquinic acid compared to the raw vegetable, where the enzymatic processes could have occurred prior to analysis [54]. Mediani et al. [51] reported that a reduction in phenolic compounds after drying might be due to oxidative degradation.

#### 3.4.3. Characterization of Flavonoid Derivatives

Peaks 9, 28, 42, and 32–36, with precursor ions 707, 625, 781, 639, 711, 609, 463, and 771 *m*/*z*, respectively, were classified as flavanols. Peak 9 was identified as kaempferide 3-rhamnoside-7-(6”-succinylglucoside), peak 28 was identified as quercetin-3-glucosyl-(1-2)-galactoside, and peak 42 as kaempferol 3-(2-”rhamnosyl-6”-acetyl-galactoside)-7-rhamnoside. Peak 32 was identified as isorhamnetin 3-glucosyl-(1-2)-galactoside, while peak 33 was identified as quercetin-O-malonylglucoside-o-glycoside. Peak 34 was identified as quercetin 3-O-robinobioside, peak 35 was identified as quercetin-3-galactoside, and peak 36 was identified as kaempferol-o-glucoside-o-sophoroside. Kaempferide 3-rhamnoside-7-(6”-succinylglucoside) and isorhamnetin 3-(2-”rhamnosyl-6”acetyl-galactoside)-7-rhamnoside were negatively affected by cooking and drying. Substantially low kaempferide 3-rhamnoside-7-(6”-succinylglucoside) concentration was observed for this study for all the cooking and drying methods (0.4–60.7 mg/kg) with the control sample having the highest concentration (60.7 mg/kg) while a concentration of 1.8–50.5 mg/kg was observed for peak 32 with the control being the highest.

The thermal degradation of the flavonoid content after drying, blanching, steaming the vegetables has been reported by various authors [34,55]. This loss could be due to oxidative degradation in the case of drying, nutrient leaching with regard to blanching, and kaempferide 3-rhamnoside-7-(6”-succinylglucoside) and isorhamnetin 3-(2-”rhamnosyl-6”acetyl-galactosiede)-7-rhamnoside destruction by heat with to respect frying and steaming, since cooking temperatures were above 100 °C [4,55].

Quercetin-3-O-robinobioside concentration was increased by different treatment methods. The steamed sample exhibited the highest flavonol concentration (106.2 mg/kg). This compound is reported to possess anti-tumor and antioxidant properties [56]. Quercetin-3-galactoside, also known as hyperoside, possesses antidiabetic, anti-inflammatory, antioxidant, and antithrombotic properties [57]. This flavanol was negatively affected by heat with the control having a concentration of 104.6 mg/kg.

**Figure 3:**
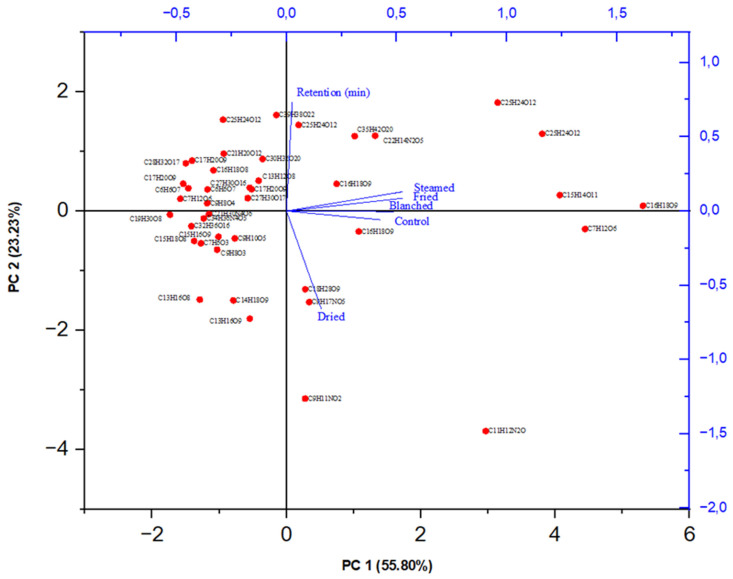
Biplot of principal component PC1 versus PC2 loadings for *C. olitorius* leaves’ metabolites.

Using principal component analysis (PCA), the cumulative contribution of principal component 1 (PC1) and principal component 2 (PC2) was 79.03%, with 55.80% attributed to PC1 and 23.23% attributed to PC2 (Figure 3).

The control, steamed, blanched, and fried samples are clustered together and are associated with PC1. These cooking methods seemed to be related as they are closely positioned to each other, demonstrating low variations within the dataset. They were characterized by high concentrations of Neochlorogenic acid (C_16_H_18_O_9_) (173.0–209.8 mg/kg), 2-O-Caffeoylhy- droxycitric acid (C_15_H_14_O_11_) (307.1–450.8 mg/kg), Quinic acid (C_7_H_12_O_6_) (338.9–416.2 mg/kg), 3,5-dicaffeoylquinic acid (C_25_H_24_O_12_) (312.9–398.1 mg/kg), and Kaempferol-o-glycoside-o-sophoroside (C_22_H_14_N_2_O_5_) (161.6–229.8 mg/kg), as also shown in Table 3. On the other hand, metabolites loaded on PC2 were associated with high retention time. Metabolites that were closely positioned to each other exhibited insignificant variations among each other in terms of retention time. It appears that the high retention of metabolites significantly influenced their concentration, as observed in 4,5-dicaffeoylquinic acid (C_25_H_42_O_12_) and 3,5-dicaffeoylquinic acid (C_25_H_42_O_12_). Among the metabolites loaded in PC2, 4,5-dicaffeoylquinic acid (C_25_H_42_O_12_) and 3,5-dicaffeoylquinic acid (C_25_H_42_O_12_) had the highest retention times (20.3 and 19.3 min, respectively) while both showed the highest content (362.8 and 398.1 mg/kg, respectively) in the blanched sample. The drying method was loaded in PC3, which had an insignificant contribution towards variation in the data set; as a result, the data in this PC was considered as noisy data and discarded.

## 4. Conclusions

Boiling improves the crude protein and crude fibre content of *Corchorus olitorius* leaves compared to the other cooking and drying methods. However, it results in the least antioxidant activity when determined with DPPH and FRAP assays. The highest increase in TPC and TFC is achieved with steaming *C. olitorius* leaves, as well as having the highest antioxidant activity. With LC-MS, the highest concentration of phytochemical compounds occurred with blanching and steaming, while the least occurred with dried *C. olitorius* leaves. Among phenolic acids, crypto-chlorogenic acid was the most abundant in blanched, steamed, and fried *C. olitorius* leaves. With flavonoids, quercetin derivatives were the most abundant compounds and their concentration was high in steamed *C. olitorius*. Overall, steaming appears to be the best cooking method for *C. olitorius*, with respect to phytochemical content and antioxidant activity. As *C. olitorius* is still categorized as an underutilized plant, the findings of this research may encourage its usage as a complement to starchy diets and its commercialization as a functional food product.

## Figures and Tables

**Table 1:** Effect of cooking and drying on TPC and TFC of *C. olitorius* leaves.

Sample Type	TPC (mg GAE/g)	TFC (mg QE//g)
Control	47.40 ^a^ ± 1.02	25.50 ^a^ ± 0.86
Blanched	88.35 ^c^ ± 1.76	36.65 ^c^ ± 3.33
Boiled	52.93 ^a^ ± 5.79	39.47 ^c^ ± 1.18
Dried	99.02 ^d^ ± 2.37	44.57 ^d^ ± 4.29
Fried Olive Oil	106.86 ^e^ ± 3.66	31.75 ^b^ ± 1.37
Fried Vegetable Oil	80.64 ^b^ ± 1.62	55.31 ^e^ ± 1.20
Steamed	118.89 ^f^ ± 3.32	78.42 ^f^ ± 2.59

Means ± standard deviation; mean values followed by different superscript letters in the same column are significantly different at *p* < 0.05; results expressed in dry matter weight (dw).

**Table 2:** Effect of cooking and drying on the free radical scavenging activity of *C. olitorius*.

Sample Type	DPPH (µM TE/g)	FRAP (µM TE/g)	ABTS (µM TE/g)
Control	66.46 ^b^ ± 0.30	139.24 ^b^ ± 10.12	75.49 ^a^ ± 2.89
Blanched	82.28 ^d^ ± 0.35	146.61 ^d^ ± 2.24	95.30 ^c^ ± 6.67
Boiled	56.70 ^a^ ± 0.66	72.62 ^a^ ± 0.17	107.29 ^d^ ± 0.58
Dried	87.77 ^e^ ± 0.47	151.69 ^e^ ± 5.77	111.20 ^e^ ± 0.73
Fried Olive Oil	99.12 ^f^ ± 1.40	159.96 ^f^ ± 6.29	119.45 ^f^ ± 3.00
Fried Vegetable Oil	70.24 ^c^ ± 10.12	143.22 ^c^ ± 7.44	81.89 ^b^ ± 5.72
Steamed	119.58 ^g^ ± 4.47	167.31 ^g^ ± 9.23	122.23 ^g^ ± 5.77

Means ± standard deviation; mean values followed by different superscript letters in the same column are significantly different at *p* < 0.05; results expressed in dry matter weight.

**Table 3:** Metabolites detected in *C. olitorius* leaves after blanching, drying, frying with olive oil, and steaming using LC-MS.

Molecule No.	Metabolite Name	Retention Time/min	Average *m*/*z*	Molecular Formula	Control	Blanched (mg/kg)	Dried (mg/kg)	Fried (mg/kg)	Steamed (mg/kg)
1	L-Phenylalanine	6.8	164	C_9_H_11_NO_2_	110.6	120.9	184.9	102.6	115.0
2	Pseudolaroside A	7.1	299	C_13_H_16_O_8_	52.8	42.7	47.3	29.7	37.1
3	Gentesic acid 5-O-glucoside	7.9	315	C_13_H_16_O_9_	110.3	85.7	86.4	57.1	60.7
4	Pantothenic acid	8.0	218	C_9_H_17_NO_5_	25.9	50.5	67.0	42.8	47.0
5	1-O-vanilloyl-beta-D-glucose	8.5	329	C_14_H_18_O_9_	80.4	69.7	72.5	51.1	62.4
6	L-Tryptophan	9.3	203	C_11_H_12_N_2_O	285.3	264.1	278.1	232.3	251.5
7	Neochlorogenic acid	9.5	353	C_16_H_18_O_9_	173.0	194.6	9.6	189.8	209.8
8	Aesculin	10.5	339	C_15_H_16_O_9_	79.7	55.7	17.1	47.7	61.4
9	Kaempferide 3-rhamnoside-7-(6”-succinylglucoside)	10.5	707	C_32_H_36_O_16_	60.7	30.3	0.4	43.5	22.1
10	4-Hydroxybenzoic acid	10.7	137	C_7_H_6_O_3_	71.6	43.8	26.1	27.6	30.8
11	1-O-p-Coumaroyl-beta-D-glucose	11.8	325	C_15_H_18_O_8_	48.9	29.0	41.3	29.4	29.3
12	4-Hydroxycinnamic acid	11.8	163	C_9_H_8_O_3_	60.5	49.7	55.3	48.4	52.2
13	Quinic acid	11.9	191	C_7_H_12_O_6_	338.9	405.9	63.7	393.2	416.2
14	Crypto-chlorogenic acid	11.9	353	C_16_H_18_O_9_	65.2	601.9	68.8	563.6	580.8
15	2-O-Caffeoylhy- droxycitric acid	12.0	69	C_15_H_14_O_11_	450.8	307.1	5.9	402.9	315.9
16	Caffeic acid	12.6	179	C_9_H_8_O_4_	71.7	35.6	4.1	57.1	42.9
17	5-Hydroxy-6-methoxycoumaric acid	13.0	369	C_7_H_12_O_6_	32.0	26.1	3.2	27.4	27.4
18	Chlorogenic acid	13.4	353	C_16_H_18_O_9_	120.2	196.2	6.7	185.6	186.4
19	7-Epi-12-hydroxyjasmonic acid glucoside	13.6	387	C_18_H_28_O_9_	148.5	132.4	139.6	82.8	106.6
20	Oxalosuccinic acid	14.0	189	C_6_H_6_O_7_	28.2	31.7	6.6	43.3	35.6
21	Citroside A	14.1	431	C_19_H_30_O_8_	20,1	6.0	40.6	8.2	11.1
22	Caffeoylmalic acid	14.1	295	C_13_H_12_O_8_	110.7	81.3	2.2	102.7	99.0
23	Syringic acid	14.7	197	C_9_H_10_O_5_	39.2	79.1	89.3	56.0	78.0
24	3-hydroxy-2-(3-methyl-2-{[3-oxo-2-(propan-2-yl)-1,2,3,4-tetrahydroquinoxaline-1-carbonyl]amino}butanamido)butanoic acid	14.9	433	C_21_H_30_N_4_O_6_	59.4	33.4	54.4	44.0	37.6
25	N-(3-butoxypropyl)-2-[10-(4-methoxyphenyl)-12,14-dioxo-8,11,13-triazatetracyclo-hexadeca-1(9),2,4,6-tetraen-13-yl]benzamide	15.0	579	C_34_H_36_N_4O5_	29.9	37.9	63.6	41.0	43.9
26	3-O-Caffeoyl-4-O-methylquinic acid	15.0	367	C_17_H_20_O_9_	40.6	28.0	13.2	20.9	23.1
27	Oxalosuccinic acid	15.2	189	C_6_H_6_O_7_	50.8	43.6	27.1	50.1	46.5
28	Quercetin 3-glucosyl-(1-2)-galactoside	15.2	625	C_27_H_30_O_17_	60,1	100.9	43.8	75.7	94.9
29	3-O-Caffeoyl-4-O-methylquinic acid	15.3	367	C_17_H_20_O_9_	98.9	95.9	30.4	73.2	83.2
30	3-O-p-Coumaroylquinic acid	15.4	337	C_16_H_18_O_8_	74.0	49.6	3.9	45.8	58.0
31	3-O-Caffeoyl-4-O-methylquinic acid	16.4	367	C_17_H_20_O_9_	49.2	37.7	4.7	29.8	32.2
32	Isorhamnetin 3-glucosyl-(1-2)-galactoside	16.3	639	C_28_H_32_O_17_	50.5	42.1	5.2	1.8	33.5
33	Quercetin-O-malonylglucoside-o-glycoside	16.5	711	C_30_H_32_O_20_	72.9	119.5	13.0	108.1	105.3
34	Quercetin 3-O-robinobioside	16.5	609	C_27_H_30_O_16_	48.6	100.8	51.8	76.6	106.2
35	Quercetin 3-galactoside	17.6	463	C_21_H_20_O_12_	104.6	75.9	12.6	27.8	48.9
36	Kaempferol-o-glycoside-o-sophoroside	18.53	771	C_22_H_14_N_2_O_5_	191.7	229.8	19.7	161.6	226.6
37	Tricin 7-[feruloyl-(2)-glucuronyl-(1-2)-glucuronide]	19.3	857	C_39_H_38_O_22_	5.91	153.9	9.2	75.4	231.4
38	1,4-dicaffeoylquinic acid	18.7	515	C_25_H_24_O_12_	123.4	56.1	7.9	166.4	172.8
39	3,5-dicaffeoylquinic acid	19.3	515	C_25_H_24_O_12_	312.9	398.1	44.5	313.7	379.8
40	1,3-dicaffeoylquinic acid	19.5	515	C_25_H_24_O_12_	50.3	74.8	3.2	55.6	81.3
41	4,5-dicaffeoylquinic acid	20.3	515	C_25_H_24_O_12_	223.8	362.8	20.1	341.4	333.5
42	Kaempferol 3-(2”-rhamnosyl-6”-acetylgalactoside) 7-rhamnoside	21.6	781	C_35_H_42_O_20_	59.3	266.1	75.0	175.3	208.3

## Data Availability

Not applicable.

## Sample Availability

Samples of the compounds are not available from the authors.
